# Validation and reliability of the Italian versions of the Food Allergy Quality of Life ‐Parental Burden (FAQL‐PB) and ‐Parent Form 10 (FAQLQ‐PF10) Questionnaires

**DOI:** 10.1111/pai.70510

**Published:** 2026-10-02

**Authors:** Pasquale Comberiati, Simona Barni, Mario Miccoli, Audrey DunnGalvin, Francesca Mori, Lucrezia Sarti, Andrea Poli, Daniel Munblit, Enza D'Auria, David Fleischer, Diego G. Peroni, Mattew Greenhawt

**Affiliations:** ^1^ Department of Clinical and Experimental Medicine, Section of Pediatrics University of Pisa Pisa Italy; ^2^ Allergy Unit, Department of Pediatrics Meyer Children's University Hospital Florence Italy; ^3^ Department of Human and Social Sciences Università Mercatorum Rome Italy; ^4^ School of Applied Psychology University College Cork Cork Ireland; ^5^ Inflammation, Repair and Development Section, NHLI Imperial College London London UK; ^6^ Department of Biomedical and Clinical Sciences University of Milan Milan Italy; ^7^ Allergy Unit, Department of Pediatrics Buzzi Children's Hospital Milan Italy; ^8^ Department of Pediatrics, Section of Allergy/Immunology, Children's Hospital Colorado University of Colorado School of Medicine Aurora Colorado USA; ^9^ Aquestive Therapeutics, Inc Warren New Jersey USA

**Keywords:** caregivers, children, FAQL‐PB, FAQLQ‐PF10, food allergy, quality of life, questionnaires

## Abstract

**Background:**

Food allergy (FA) may substantially impair quality of life (QoL) in affected children and their families. Reliable and culturally adapted tools are needed to assess this burden. We aimed to translate, culturally adapt, and validate the Italian versions of the Food Allergy Quality of Life Questionnaire Parent Form 10 (FAQLQ‐PF10) and the Food Allergy Quality of Life Parental Burden Questionnaire (FAQL‐PB).

**Methods:**

Following World Health Organization recommendations, the questionnaires underwent forward–backward translation and psychometric validation. Internal consistency, test–retest reliability, convergent validity, and discriminant validity were assessed in caregivers of children with FA (*n* = 128) and without FA (*n* = 52).

**Results:**

Both questionnaires showed high internal consistency (Cronbach's *α* = 0.87 for FAQLQ‐PF10 and 0.95 for FAQL‐PB) and excellent test–retest reliability (Intraclass Correlation Coefficients 0.95 and 0.87, respectively). Moderate correlations with the Food Allergy Independent Measure (FAIM) supported convergent validity, whereas weak correlations with generic QoL and psychological distress measures confirmed disease specificity. FAQLQ‐PF10 and FAQL‐PB scores were significantly higher in the FA group than in controls (all *p* < .0001), supporting discriminant validity. Key clinical predictors of worse QoL included history of anaphylaxis, allergic comorbidities, and previous emergency department visits for FA. Parents of children with milk or egg allergy were a high‐risk subgroup for impaired QoL, whereas increasing child age, longer elimination diet duration, and multiple affected siblings were associated with worse child QoL.

**Conclusions:**

The Italian versions of the FAQLQ‐PF10 and FAQL‐PB are valid, reliable, and disease‐specific instruments for assessing FA‐related QoL. Their use may facilitate routine QoL assessment and support patient‐centered management of pediatric FA.

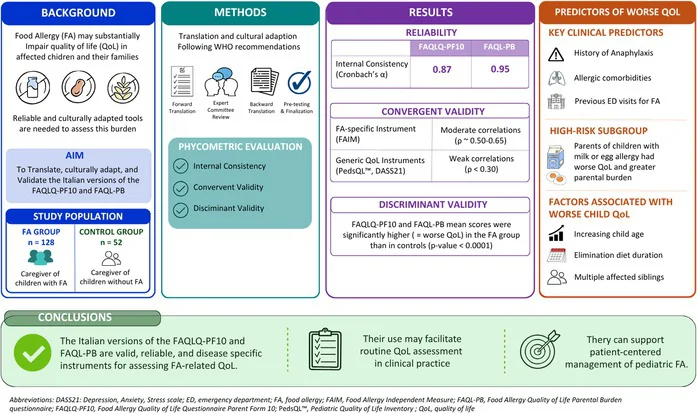

## Graphical abstract

The Italian versions of the FAQLQ‐PF10 and FAQL‐PB demonstrated excellent reliability, validity, and disease specificity in assessing food allergy‐related quality of life. Their use may facilitate routine assessment of the burden of pediatric food allergy and support more patient‐centered management.

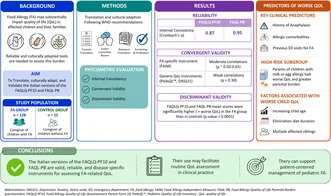

**Abbreviations**
DASS21: Depression, Anxiety, Stress scale
ED: emergency department
FA: food allergy
FAIM: Food Allergy Independent Measure
FAQL‐PB: Food Allergy Quality of Life Parental Burden Questionnaire
FAQLQ‐PF10: Food Allergy Quality of Life Questionnaire Parent Form 10
ICCs: Interclass Correlation Coefficients
MD: mean difference
PedsQL™: Pediatric Quality of Life Inventory
QoL: quality of life

**Key message**

This is the first study validating the Food Allergy Quality of Life Questionnaire Parent Form 10 (FAQLQ‐PF10) and the Food Allergy Quality of Life Parental Burden Questionnaire (FAQL‐PB) in the Italian population. The Italian versions of these two questionnaires demonstrated excellent reliability and validity for assessing food allergy‐related quality of life. These brief disease‐specific instruments may support routine clinical evaluation and help identify children and families at increased risk of quality‐of‐life impairment.

## 1 INTRODUCTION

Food allergy (FA) represents a growing public health concern worldwide, affecting individuals across all age groups.^1^ Epidemiological studies have documented increasing incidence and prevalence of FA, together with rising rates of hospitalization for food‐induced anaphylaxis among Italian children.^2^, ^3^

FA remains the leading cause of anaphylactic reactions in the pediatric population.^4^, ^5^ Although several novel therapeutic approaches are emerging, the cornerstone of FA management remains strict dietary avoidance of culprit foods and use of self‐administrable epinephrine in case of anaphylaxis.^6^ However, maintaining an elimination diet is challenging because many common allergens are widely used in processed foods. Moreover, the need for constant vigilance and preparedness for accidental exposures can affect multiple domains of daily life, such as school attendance, social activities, and family life.^7^, ^8^

Not surprisingly, FA has been shown to strongly impair children's social interactions, emotional states, and quality of life (QoL).^8^, ^9^ There is also evidence of long‐term psychosocial distress after resolution of FA, when approximately 20%–30% of previously food‐allergic children continue an elimination diet due to food refusal or fear of allergy persistence.^9^ Early studies showed that the diagnosis of FA significantly also impairs parental QoL due to the stress and anxiety associated with continuous vigilance for strict allergen avoidance and the threat of anaphylaxis from inadvertent exposures.^10^, ^11^

There is increasing recognition that health‐related QoL is a key outcome in pediatric allergy research and clinical care. Generic QoL instruments, while useful for broad comparisons, often lack sensitivity to capture disease‐specific challenges such as fear of accidental exposure, daily life limitations, or dietary restrictions. Previous research has demonstrated that FA‐related QoL is influenced by a psychosocial burden and driven by factors such as uncertainty, vigilance, perceived risk, family adaptation, and various social determinants, many of which are not captured by traditional clinical outcomes.^12^

As a result, FA‐specific instruments have been developed to better assess the unique burden associated with this condition.^13^ Among these, the Food Allergy Quality of Life Parental Burden Questionnaire (FAQL‐PB) specifically measures the parental burden of illness,^14^ whereas the Food Allergy Quality of Life Questionnaire Parent Form (FAQLQ‐PF) assesses the child's QoL as reported by parents.^15^ A shorter version, the FAQLQ‐PF10, has been developed to provide a rapid yet comprehensive assessment suitable for clinical practice.^16^ Although the extended version of the FAQLQ‐PF has been previously translated and validated in Italian populations,^17^ no validated Italian versions of the shorter FAQLQ‐PF10 or the FAQL‐PB are currently available. This represents a significant limitation given the need for brief, reliable tools that can be easily implemented in routine care. Furthermore, cultural and linguistic differences may influence how caregivers perceive and report the burden of FA,^18^ highlighting the importance of proper cross‐cultural adaptation and validation of these instruments.

The primary aim of this study was to translate, culturally adapt, and validate the Italian versions of the FAQL‐PB and FAQLQ‐PF10 Questionnaires. The secondary aim was to evaluate the discriminant validity of these tools and to identify clinical factors associated with impaired FA‐related QoL in children with FA and their caregivers.

## 2 METHODS

### 2.1 Translation and cross‐cultural adaptation of the FAQLQ‐PF10 and the FAQL‐PB questionnaires into Italian

Translation and cross‐cultural adaptation of the FAQL‐PB and FAQLQ‐PF10 followed the World Health Organization (WHO) recommendations^19^ (Appendix S1). Content validity was assessed through expert review. Face validity was evaluated in a pilot pre‐test involving parents of children with FA, who assessed the comprehensibility, clarity, acceptability, and relevance of the translated items. Participants' feedback was incorporated to refine the wording before the Italian versions were finalized. The final versions of the questionnaires were administered to the target population for psychometric evaluation. Test–retest reliability was assessed in a randomly selected subset of participants, who completed the questionnaires twice, 1 week apart, using intraclass correlation coefficients (ICCs).

### 2.2 Study design

Between February 1, 2019 and December 1, 2020, a total of 180 caregivers were consecutively recruited at the pediatric allergy unit of two Italian university hospitals (i.e., the Azienda Ospedaliero Universitaria Pisana AOUP in Pisa, and the Meyer Children's Hospital in Florence); 128 had a child (0–17 years) diagnosed with IgE‐mediated FA (active group), and 52 were parents of age‐matched children without FA or chronic diseases (control group). In the active group, the diagnosis of FA was established either on the basis of a clear history of an immediate allergic reaction (including anaphylaxis) associated with evidence of IgE‐mediated sensitization to the culprit food (defined as skin prick test wheal diameter ≥3 mm greater than the negative control and/or serum food‐specific IgE levels ≥0.35 kUA/L) or by a positive oral food challenge in children with an equivocal clinical history and evidence of IgE‐mediated sensitization to the suspected food allergen. Children in the control group were referred to the pediatric allergy clinic for conditions unrelated to FA, including suspected respiratory allergy, acute viral urticaria, or non‐allergic skin rashes. None had suspected or confirmed FA, food avoidance, or dietary restrictions nor a chronic disease. The study was approved by the Local Institutional Review Board (n.831/19). Written informed consent was obtained from all participants.

### 2.3 Data collection and measures

At the screening visit, caregivers who provided informed consent completed a questionnaire collecting demographic information and the child's allergy history. Clinical information was subsequently verified through medical chart review. In addition to the FAQL‐PB and FAQLQ‐PF10 Questionnaires, parents completed three validated instruments to assess convergent validity: (1) the Pediatric Quality of Life Inventory (PedsQL™, a generic pediatric QoL instrument; normal values ≥80),^20^ (2) the Depression Anxiety Stress Scales (DASS21, a generic screening questionnaire assessing depression, anxiety, and stress in adults; normal values ≤9 for depression, ≤7 for anxiety, ≤14 for stress),^21^ and (3) the Food Allergy Independent Measure (FAIM, a disease‐specific instrument that assesses parental expectations regarding FA‐related outcomes).^17^ The FAQLQ‐PF10 was restricted to caregivers of children aged 0–12 years, consistent with its original validation,^13^ whereas the FAQL‐PB Questionnaire was completed by all participants.

The FAQLQ‐PF10 is an age‐appropriate parent‐proxy measure that assesses the impact of everyday social and dietary limitations and measures the emotional impact of such restrictions on the lives of children ≤12 years with FA. It has demonstrated excellent reliability (Cronbach's *α* = .89) and evidence of construct, cross‐cultural content, and longitudinal validity.^16^, ^22^ The questionnaire consists of 10 items rated on a 7‐point Likert scale.^16^ Previous factor analyses have identified three factor‐derived subdomains: Emotional Impact, Food Anxiety, and Social and Dietary Limitations (Appendix S2).^23^ In this study, only the FAQLQ‐PF10 total score, calculated as the mean of all 10 items according to the validated scoring procedure, was used for the analyses. Higher scores indicate poorer FA‐related QoL.

The FAQL‐PB is a self‐administered questionnaire that measures the impact of pediatric FA on caregiver QoL. It was developed by Cohen et al.,^14^ who reported a Cronbach α of 0.95 and an intraclass correlation of 0.93, indicating both excellent validity and reliability for the cross‐sectional assessment of QoL. The questionnaire was subsequently validated by Ward and Greenhawt,^24^ who reported a 2‐factor structure and a Minimal Clinically Important Difference (MCID) of 0.3. The FAQL‐PB consists of 17 items rated on a 7‐point Likert scale. Factor analyses have identified two factor‐derived subdomains, namely Limitations on Life assessing restrictions in family and social activities related to the child's FA, and Emotional Distress, assessing parental worry, anxiety, and emotional burden associated with managing the child's FA (Appendix S2).^25^ In this study, only the FAQL‐PB total score, calculated as the mean of all 17 items according to the validated scoring procedure, was used for the analyses. Higher scores indicate worse QoL.

### 2.4 Statistical analysis

Post‐hoc power analyses were performed for all primary significant results, using 0.80 as the minimum acceptable threshold; this target was met across all primary analyses. Internal consistency was assessed using Cronbach's alpha coefficients for total scores and subdomains. Construct validity was evaluated through convergent and discriminant validity analyses. Convergent validity was evaluated by calculating Spearman correlation coefficients between translated questionnaires and both generic and disease‐specific QoL instruments. Discriminant validity was assessed by comparing participants with and without FA using an unpaired *t*‐test.

Categorical variables were summarized as percentages, and associations between categorical variables were assessed using the chi‐square test. Quantitative variables were reported as mean and standard deviation (SD), based on their distribution as assessed by the Shapiro–Wilk test for normality. Differences between the two independent groups were evaluated using the *t*‐test and with chi‐squared test or Fisher's exact test (depending on frequencies).^26^ Logistic ordinal regression analyses were performed to estimate univariate and multivariable regression coefficients with 95% confidence intervals (CIs) and *p*‐values. All analyses were conducted using the open‐source software R (https://www.r‐project.org/) and SPSS® 25 (IBM Corp., Armonk, NY, USA), with statistical significance set at *p* < .05.

## 3 RESULTS

### 3.1 Participant characteristics

Baseline characteristics of participants are reported in Table 1, showing no significant differences between groups in terms of age, sex distribution, caregiver characteristics, socioeconomic status, and DASS21 and PedsQL™ scores. More than two‐thirds of respondents were mothers, whereas most children were male. Among children with FA, the most common allergens were milk (43%) and egg (33%), followed by peanut and tree nuts (Table 2). A large proportion of patients (58%) had multiple food allergies. Almost half of the cohort had experienced anaphylaxis, and 34% had emergency department (ED) visits due to food allergic reactions, with less than 10% having used or received intramuscular epinephrine. Additionally, 65% had at least one other atopic comorbidity.

**TABLE 1:** Baseline characteristics.

Characteristics	Food allergy (*n* = 128)	Non‐food allergy (*n* = 52)
Children age	7.45 ± 4.4	7.42 ± 4.7
Children age groups		
0–3 years	38 (30)	16 (31)
4–6 years	30 (23)	9 (17)
7–12 years	44 (34)	18 (35)
13–17 years	16 (13)	9 (17)
Children sex (male)	80 (62.5)	31 (60)
≥1 sibling	83 (66)	34 (65)
Other siblings with food allergy	8 (6)	1 (2)
Caregiver filling questionnaires (mother)	101 (79)	38 (73)
Caregiver age	41.7 ± 9.2	40.8 ± 5.7
Caregiver white race	126 (98)	50 (96)
Caregiver education level		
Middle School	10 (8)	8 (16)
High School	69 (55)	22 (43)
Bachelor	29 (23)	17 (33)
Master	17 (14)	4 (8)
Caregiver marital status		
Unmarried	23 (18)	12 (23)
Married	91 (73)	36 (71)
Divorced	9 (7)	3 (6)
Widowed	2 (2)	0 (0)
Household income (Euros/year)		
<25,000	26 (24)	17 (36)
25,000–50,000	57 (53)	23 (49)
50,000–100,000	20 (19)	6 (13)
>100,000	4 (4)	1 (2)
Caregiver chronic diseases	25 (20)	12 (23)
Caregiver DASS21 Depression	7.75 ± 11.6	7.46 ± 11.0
Caregiver DASS21 Anxiety	6.1 ± 11.1	6.6 ± 9.8
Caregiver DASS21 Stress	17.7 ± 15.3	18.6 ± 16.3
PedsQL™ Total Score	85.3 ± 12.1	87.7 ± 7.7
FAIM Total Score	3.1 ± 1.1	0.5 ± 0.3
FAQL‐PB Total Score	2.8 ± 1.4	0.0 ± 0.0
FAQLQ‐PF10 Total Score	2.7 ± 1.3	0.0 ± 0.1

*Note*: Shown are the number (and %) for categorical variables and mean ± standard deviation for continuous variables.

**TABLE 2:** Characteristics of children with food allergy.

Food allergens	
Milk	55 (43)
Egg	42 (33)
Peanut	32 (25)
Hazelnut	36 (28)
Walnut	37 (29)
Other Tree Nuts	27 (21)
Fish	15 (12)
Shellfish	3 (2)
Other Foods	36 (29)
Multiple food allergy	74 (58)
No of foods avoided due to allergy	2.3 ± 1.6
Age at food allergy diagnosis	2.4 ± 3.0
Years of Elimination Diet	5.0 ± 4.0
Most Severe Reaction involved	
Skin	110 (86)
Gastrointestinal Tract	85 (66)
Upper Respiratory Tract	69 (54)
Lower Respiratory Tract	46 (36)
Cardiovascular	22 (17)
Anaphylaxis	55/122 (45)
History of Epinephrine use for food allergy	10/124 (8)
Emergency Department visits for food allergy	40/117 (34)
Oral Food Challenge in the past	59 (46)
Other Atopic Comorbidities	81/125 (65)
Asthma	28/125 (22)
Allergic Rhinitis	31/125 (25)
Atopic Dermatitis	48/125 (38)
Food allergy Advocacy Group Membership	13 (10)

*Note*: Shown are the number (and %) for categorical variables and mean ± standard deviation for continuous variables.

### 3.2 Test–retest reliability, internal consistency, and convergent validity

ICCs between the questionnaires completed twice, at a 1‐week interval, were 0.95 (95% CI 0.87–0.98; *p* < .0001) for the FAQLQ‐PF10 and 0.87 (95% CI 0.67–0.95; *p* < .0001) for the FAQL‐PB, indicating high test–retest reliability.

Cronbach's α was 0.87 for the FAQLQ‐PF10 and 0.95 for the FAQL‐PB, demonstrating high internal consistency. Similar results were shown for the exploratory factor‐derived subdomains of both questionnaires (Appendix S2; Table S1), indicating adequate reliability across domains.

Moderate correlations were observed between the two FAQL Questionnaires and between each questionnaire and the FAIM, supporting convergent validity (Tables S2–S7). Specifically, the FAQLQ‐PF10 mean total score correlated moderately with both the FAQL‐PB mean total score (*ρ* = .493, *p* < .01) and the FAIM (*ρ* = .550, *p* < .01). Similarly, the FAQL‐PB mean total score showed a moderate correlation with the FAIM (*ρ* = .657, *p* < .01).

In contrast, correlations with generic QoL measures were weak. The FAQL‐PB mean total score showed only a weak negative correlation with the PedsQL™ total score (*ρ* = −.264, *p* < .01), whereas the correlation between the FAQLQ‐PF10 mean total score and the PedsQL™ was not statistically significant (*ρ* = −.162, *p* = ns). Likewise, both FAQL Questionnaires showed only weak correlations with DASS‐21 scores, indicating that these instruments capture aspects of FA‐related QoL that are not adequately reflected by generic QoL or psychological distress measures (Tables S2–S7).

### 3.3 Discriminant validity and predictors of poorer QoL

Both mean total scores of the FAQL‐PB and the FAQLQ‐PF10 were significantly higher in children with FA than in controls (*p* < .0001; Table S8), confirming strong discriminant validity. In contrast, no significant between‐group differences were observed for the generic PedsQL™ or DASS‐21 scores (Table 1), further supporting the disease‐specific nature of the FAQL Questionnaires.

Univariate regression analysis identified common and questionnaire‐specific predictors of worse FA‐related QoL (Table S9). A history of anaphylaxis emerged as a common predictor of higher (worse) total scores on both the FAQLQ‐PF10 and the FAQL‐PB (Figure 1). Likewise, children who had required an ED visits for FA had significantly higher mean total scores than those without a previous ED visit on both the FAQLQ‐PF10 (3.25 ± 1.32 vs. 2.57 ± 1.38; mean difference (MD) = 0.68; *t* = 2.54, Cohen's d = 0.504; *p* = .012) and the FAQL‐PB (3.14 ± 1.39 vs. 2.57 ± 1.48; MD = 0.57; *t* = 2.01, Cohen's d = 0.39; *p* = .047). Similarly, children with allergic comorbidities had significantly higher FAQLQ‐PF10 mean total scores than those with isolated FA (2.93 ± 1.36 vs. 1.14 ± 1.54; MD = 1.79; *t* = 8.00, Cohen's d = 1.22; *p* < .001). A similar pattern was observed for the FAQL‐PB (2.79 ± 1.47 vs. 1.22 ± 1.59; MD = 1.57; *t* = 6.73, Cohen's d = 1.02; *p* < .001).

**FIGURE 1:**
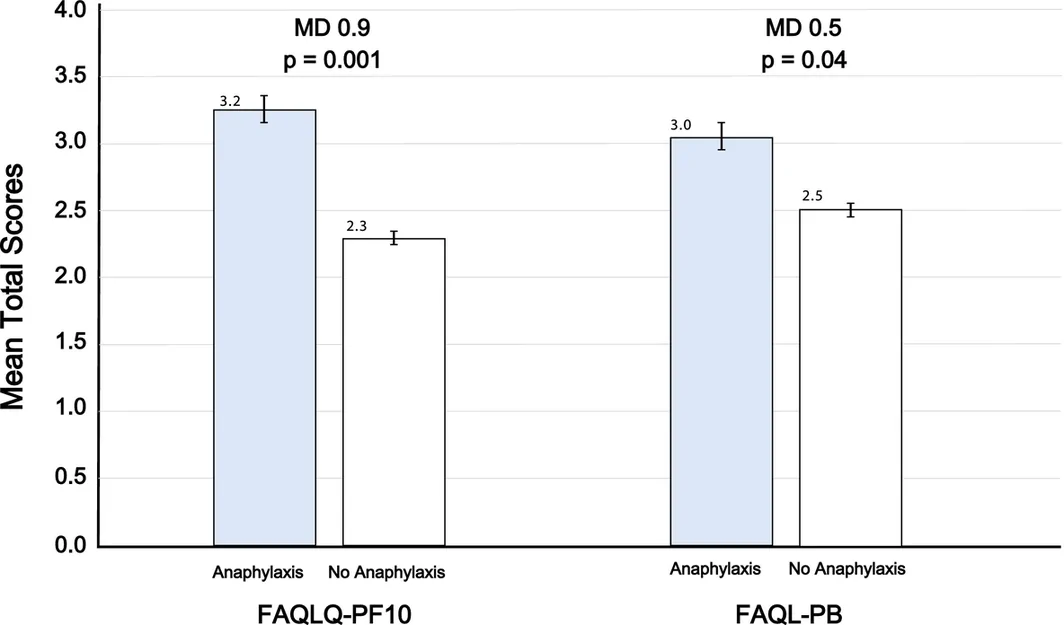
Distribution of FAQLQ‐PF10 and FAQL‐PB total scores by anaphylaxis history. Mean difference (MD) for each bar is labeled. Error bars represent standard error.

For the FAQLQ‐PF10, QoL progressively worsened with increasing child age and duration of the elimination diet (Figure 2). Children aged 0–3‐years had significantly lower mean total scores than those aged 4–6 years (1.39 ± 1.28 vs. 2.47 ± 1.86; MD = −1.08; *t* = −3.33, Cohen's d = −0.70; *p* = .001) and 7–12 years (2.15 ± 1.73; MD = −0.76; *t* = −2.66, Cohen's d = −0.50; *p* = .009). No significant difference was observed between the 4–6‐ and 7–12‐year age groups (*t* = 0.87, Cohen's d = 0.18; *p* = .385). Children with siblings affected by FA also had significantly higher FAQLQ‐PF10 mean total scores than those without affected siblings (3.42 ± 1.54 vs. 1.88 ± 1.70; MD = 1.54; *t* = 2.66, Cohen's d = 0.91; *p* = .009).

**FIGURE 2:**
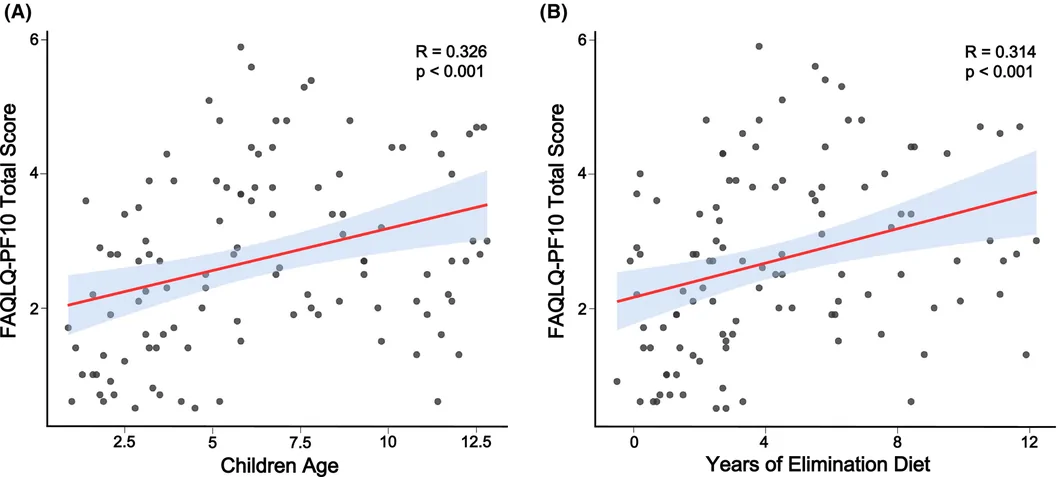
Association between FAQLQ‐PF10 total scores and age (A) and duration of the elimination diet (B).

For the FAQL‐PB, caregivers of children with milk allergy reported the highest burden (mean total score 3.25 ± 1.42; regression coefficient = 0.555, 95% CI 0.208–0.902; *t* = 3.16; *p* = .002). Furthermore, caregivers of children with milk or egg allergy reported significantly higher burden than those caring for children with peanut or tree nut allergy (Figure 3).

**FIGURE 3:**
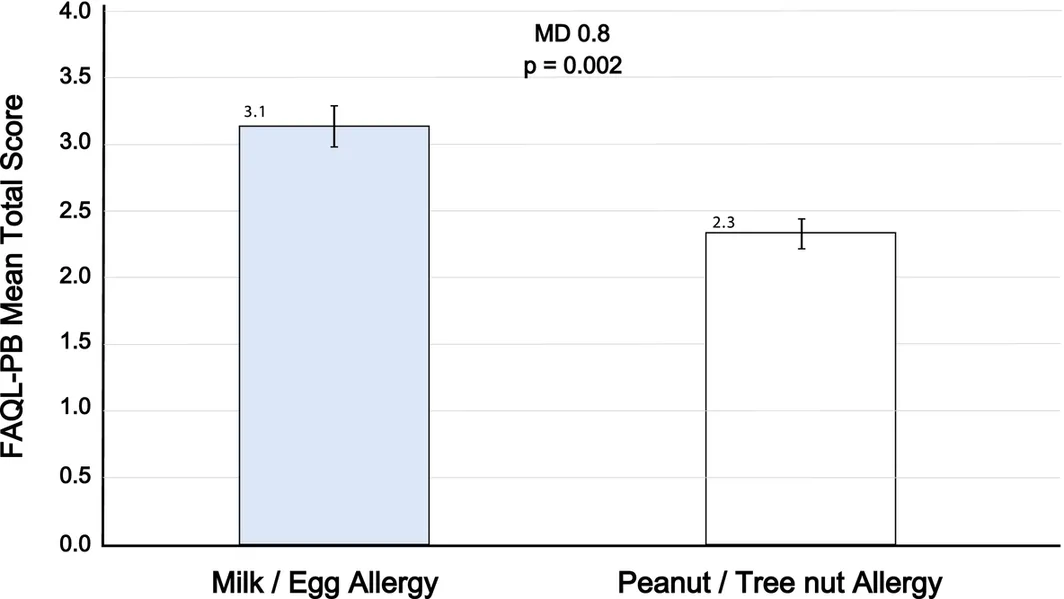
FAQL‐PB total score by allergen (milk or egg vs. peanut or tree nut). Mean difference (MD) for each bar is labeled. Error bars represent standard error.

Multivariable ordinal logistic regression confirmed these findings. For the FAQLQ‐PF10, a history of anaphylaxis (coefficient = 0.91, 95% CI 0.125–1.692; *p* = .023) and increasing child age (coefficient = 0.12, 95% CI 0.033–0.205; *p* = .006) remained independently associated with poorer QoL. For the FAQL‐PB, independent predictors of greater parental burden were a history of anaphylaxis (coefficient = 0.89, 95% CI 0.246–1.533; *p* = .007) and milk or egg allergy, compared with peanut or tree nut allergy (coefficient = 1.08, 95% CI 0.429–1.733; *p* = .001) (Table S10).

## 4 DISCUSSION

This study provides a comprehensive psychometric validation of the Italian versions of the FAQL‐PB and the FAQLQ‐PF10 Questionnaires, demonstrating that both instruments are reliable, valid, and suitable for use in clinical and research settings. History of food‐induced anaphylaxis, previous ED visits for FA, and the presence of allergic comorbidities are common predictors of impaired QoL in both food allergic children and their caregivers. In addition, increasing age, longer duration of an elimination diet, and having siblings with FA are specific factors associated with worse QoL in children, whereas caring for a child with milk or egg allergy places a greater parental burden than caring for a child with peanut or tree nut allergy.

Previous studies in Italian pediatric cohorts have mainly focused on children's QoL using the parent‐reported FAQLQ‐PF.^17^, ^27^ In contrast, our study validates the shorter FAQLQ‐PF10 for the Italian population. In addition, it is the first to validate and apply the FAQL‐PB, thereby enabling assessment of the broader impact of FA on family dynamics and parental burden.

Validating the FAQL‐PB is particularly important because the impact of FA extends beyond the affected child and often demonstrates some spillover effect of illness.^28^ Parents frequently undertake substantial responsibility for risk assessment, allergen avoidance, communication with schools and caregivers, emergency preparedness, and ongoing monitoring. Consequently, parental burden represents an important clinical outcome poorly reflected by measures focused solely on the child. This approach acknowledges that FA affects families beyond clinical outcomes, influencing daily activities, social interactions, time management, and emotional well‐being.^12^, ^13^

The psychometric properties observed in our study are consistent with those reported in the original validation studies of both FAQL‐PB and FAQLQ‐PF10 Questionnaires.^14^, ^15^, ^16^, ^24^, ^25^ In particular, the excellent internal consistency of the FAQL‐PB (*α* = .95) confirms that the questionnaire reliably measures a unified construct of parental burden. Similarly, the FAQLQ‐PF10 demonstrated good internal consistency across its domains, supporting its use as a brief yet robust tool for clinical and research purposes. The high correlation coefficients observed in the test–retest analysis further reinforce that both questionnaires provide consistent results in the absence of clinical changes, which is essential for longitudinal monitoring.

An important aspect of the validation process is the evaluation of convergent validity. The moderate correlations observed with FAIM confirm that FAQL‐PB and FAQLQ‐PF10 effectively capture disease‐specific impact. At the same time, the weak correlations with generic PedsQL™ and DASS21 highlight the limitations of generic QoL instruments in assessing FA‐related burden. The Italian version of the FAQL‐PB and the FAQLQ‐PF10 Questionnaires effectively discriminated between caregivers of children with and without FA. Of note, the absence of differences in PedsQL™ and DASS21 scores between these two groups further supports the specificity of FAQL‐PB and FAQLQ‐PF10 in identifying disease burden. These findings underscore the importance of using disease‐specific tools in both clinical practice and research.

Our findings also provide valuable clinical insights into the factors associated with impaired QoL. The association found between severe disease markers—such as anaphylaxis, ED visits, and comorbid allergic conditions—and worse QoL is in line with previous literature.^29^, ^30^, ^31^ These factors likely reflect both increased clinical severity and heightened parental anxiety. In contrast to previous American cohort studies,^29^, ^31^ the history of epinephrine use was not associated with impaired QoL in our study, which may be explained by the low number of children (8%) treated with intramuscular epinephrine.

Among children with FA, QoL impairment was found to increase with age and duration of elimination diet. These findings are in line with recent observations in an American cohort, where age emerged as a central predictor of FA‐related QoL.^31^ Deterioration of QoL with age is likely to reflect the growing awareness of dietary restrictions and social limitations as children become older and more independent. Additionally, the presence of multiple affected siblings was associated with worse QoL, suggesting a cumulative burden on family dynamics.

Among caregivers, milk allergy was the most impactful FA on QoL. This is unsurprising, considering that milk allergy is among the most frequent types of FA as well as the leading cause of hospital admission for anaphylaxis in Italian children.^2^, ^32^ Although the European anaphylaxis registry reports milk and egg as the most frequent culprits of anaphylaxis in infants, hazelnut and cashew in preschool‐aged children, and peanut at all ages, there are regional variations.^33^ In a Swedish cohort, Vetander and colleagues^34^ found that tree nuts, particularly cashew, and peanuts were the most common eliciting foods of anaphylaxis in the first 3 years of age. These reports highlight the importance of cross‐cultural adaptation and regional validation of these QoL instruments.

Finally, our study reconfirmed a US finding that peanut or tree nut allergy was associated with lower parental burden than milk or egg allergy.^29^, ^35^ This finding may reflect differences in the day‐to‐day management demands associated with these allergies. Milk and egg are ubiquitous ingredients in processed foods, home cooking, school meals, and social eating environments, making complete avoidance particularly challenging. Consequently, caregivers may experience a sustained burden of management. In contrast, although peanut and tree nut allergy are often perceived as severe, avoidance strategies may be more straightforward because these allergens are less embedded in everyday foods. This interpretation is consistent with biopsychosocial models of FA, which recognize that QoL is shaped by the interaction between clinical risk, uncertainty, environmental demands, and the ongoing burden of self‐ and family‐management.^11^, ^12^

A few limitations should be acknowledged. First, the cross‐sectional design limits causal inferences. A recall bias may result from the use of a parent‐reported questionnaire to collect demographic and clinical data. Although information was verified through chart review, any remaining recall error would most likely have introduced non‐differential misclassification, potentially attenuating rather than inflating the observed associations. In addition, participants were recruited from two university hospitals, which may have resulted in a selection bias towards children with more severe FA. Consequently, the observed burden of FA and the strength of associations with FA‐specific QoL measures may have been overestimated compared with those in the general FA population, thereby limiting the generalizability of our findings. Finally, white mothers were the predominant responders to the questionnaires, which may limit the applicability of our results to fathers and to more ethnically diverse populations. However, this is a common issue with FA QoL studies.^14^, ^29^, ^35^ Further studies including more diverse patient populations in terms of FA severity, ethnicity, and caregiver characteristics, as well as longitudinal follow‐up are needed to further elucidate predictors of FA‐related QoL and the impact of therapeutic interventions.

Despite these limitations, the rigorous methodological approach adopted for translation and validation of FAQL‐PB and FAQLQ‐PF10 in the Italian population provided robust evidence supporting their use. By strictly following the WHO recommendations, we ensured not only linguistic accuracy but also cultural relevance, which is crucial when adapting patient‐reported outcome measures across different populations. Furthermore, construct validity was supported by comparison with both generic and disease‐specific validated instruments.

In summary, the Italian versions of the FAQL‐PB and FAQLQ‐PF10 Questionnaires are valid, reliable, and clinically meaningful tools for assessing the impact of FA on children and their caregivers. The increasing incorporation of patient‐reported outcome measures into FA clinical trials and routine care highlights the need for brief, validated instruments that can be implemented across diverse healthcare and cultural settings. The availability of Italian versions of FAQLQ‐PF10 and FAQL‐PB may facilitate harmonization of QoL assessment across international studies and a more patient‐centered care. From a clinical perspective, our findings suggest that the daily management burden associated with specific allergens should be considered alongside traditional markers of disease severity. This approach may help clinicians identify families who could benefit from additional dietary, educational, or psychological support.

## AUTHOR CONTRIBUTIONS

**Pasquale Comberiati:** Conceptualization; investigation; writing – original draft; methodology; validation; writing – review and editing; formal analysis; project administration; data curation; supervision. **Simona Barni:** Investigation; writing – original draft; validation; data curation; project administration. **Mario Miccoli:** Methodology; validation; writing – original draft; software; formal analysis; supervision; data curation. **Audrey DunnGalvin:** Conceptualization; writing – review and editing; validation; visualization; supervision; resources. **Francesca Mori:** Writing – review and editing; methodology; validation; supervision; resources; data curation. **Lucrezia Sarti:** Writing – original draft; methodology; validation; data curation. **Andrea Poli:** Writing – original draft; methodology; software; formal analysis; data curation; resources. **Daniel Munblit:** Writing – review and editing; visualization; validation; methodology; supervision; resources. **Enza D'Auria:** Methodology; validation; visualization; writing – review and editing; supervision; resources. **David Fleischer:** Methodology; validation; visualization; writing – review and editing; data curation; supervision; resources. **Diego G. Peroni:** Methodology; validation; visualization; writing – review and editing; data curation; supervision; resources. **Mattew Greenhawt:** Conceptualization; writing – review and editing; methodology; validation; visualization; supervision; investigation.

## CONFLICT OF INTEREST STATEMENT

Simona Barni reports personal fees from Nutricia, Sanofi, and Firma. David Fleischer has received grant support from Aquestive, ARS Pharmaceuticals, DBV Technologies, N‐Fold; receives royalties from UpToDate; serves as a consultant to Aquestive, ARS Pharmaceuticals, DBV Technologies, Genentech, Nasus Pharma; and has stock options in Nasus Pharma and Grow Happy. Matt Greenhawt is the chief medical officer for Aquestive Therapeutics, Inc. He is a speaker for Genentech. He is a former consultant to Aquestive; speaker for ARS; advisory board member for Aquestive, DBV, Novartis, Nutricia, Genentech, ALK, Prota; former senior associate editor for Annals of Allergy, Asthma, and Immunology; served as chief medical officer for the Asthma and Allergy Foundation of America; serves as a member of the Brighton Collaboration Criteria Vaccine Anaphylaxis 2.0 working group. His work on this manuscript occurred while at Children's Hospital Colorado, but critical manuscript review and approval occurred at his current employer. His views on this manuscript are his own and not necessarily reflective of Aquestive Therapeutics, Inc. The rest of the authors declare that they have no relevant conflicts of interest.

## Supporting information


Appendix S1.

## Data Availability

The data that support the findings of this study are available on request from the corresponding author. The data are not publicly available due to privacy or ethical restrictions.

## References

[1] Islam N , Chu AWL , Sheriff F , et al. Risk factors for the development of food allergy in infants and children: a systematic review and meta‐analysis. JAMA Pediatr. 2026;180(5):486‐499. 10.1001/jamapediatrics.2025.6105 PMC12887841 41661638

[2] Nocerino R , Carucci L , Coppola S , et al. Epidemiology of Paediatric Italian food allergy: results of the EPIFA study. J Allergy Clin Immunol Glob. 2024;3(3):100246. 10.1016/j.jacig.2024.100246 PMC11035069 38655562

[3] Nocerino R , Leone L , Cosenza L , Berni Canani R . Increasing rate of hospitalizations for food‐induced anaphylaxis in Italian children: an analysis of the Italian Ministry of Health database. J Allergy Clin Immunol. 2015;135(3):833‐835.e3. 10.1016/j.jaci.2014.12.1912 25630939

[4] Baseggio Conrado A , Ierodiakonou D , Gowland MH , Boyle RJ , Turner PJ . Food anaphylaxis in the United Kingdom: analysis of national data, 1998–2018. BMJ. 2021;372:n251. doi:10.1136/bmj.n251 PMC7885259 33597169

[5] Comberiati P , Spahn J , Peroni DG . Anaphylaxis in adolescents. Curr Opin Allergy Clin Immunol. 2019;19(5):425‐431. 10.1097/ACI.0000000000000572 31318740

[6] Santos AF , Riggioni C , Agache I , et al. EAACI guidelines on the management of IgE‐mediated food allergy. Allergy. 2025;80(1):14‐36. 10.1111/all.16345 PMC11724237 39473345

[7] Westwell‐Roper C , To S , Andjelic G , et al. Food‐allergy‐specific anxiety and distress in parents of children with food allergy: a systematic review. Pediatr Allergy Immunol. 2022;33(1):e13695. 10.1111/pai.13695 34779046

[8] Knibb RC , Herbert LJ , Jones CJ , et al. Global availability and uptake of psychological services for adults, caregivers and children with food allergy. Allergy. 2024;79(10):2787‐2797. 10.1111/all.16204 39031702

[9] Gau J , Wang J . Rate of food introduction after a negative oral food challenge in the pediatric population. J Allergy Clin Immunol Pract. 2017;5(2):475‐476. 10.1016/j.jaip.2016.06.027 27566905

[10] Warren CM , Otto AK , Walkner MM , Gupta RS . Quality of life among food allergic patients and their caregivers. Curr Allergy Asthma Rep. 2016;16(5):38. 10.1007/s11882-016-0614-9 27048239

[11] Knibb RC , Howell C , Whitehouse A , Peterson CC . The role of food allergy‐related worry and self‐efficacy in explaining quality of life in caregivers of children. Pediatr Allergy Immunol. 2025;36(11):e70248. 10.1111/pai.70248 PMC12621170 41243957

[12] Protudjer JLP , Davis CM , Gupta RS , Perry TT . Social determinants and quality of life in food allergy management and treatment. J Allergy Clin Immunol Pract. 2025;13(4):745‐750. 10.1016/j.jaip.2025.02.016 40043949

[13] DunnGalvin A , Cullinane C , Daly DA , Flokstra‐de Blok BM , Dubois AE , Hourihane JO . Longitudinal validity and responsiveness of the food allergy quality of life questionnaire—parent form in children 0–12 years following positive and negative food challenges. Clin Exp Allergy. 2010;40(3):476‐485. 10.1111/j.1365-2222.2010.03454.x 20210816

[14] Cohen BL , Noone S , Muñoz‐Furlong A , Sicherer SH . Development of a questionnaire to measure quality of life in families with a child with food allergy. J Allergy Clin Immunol. 2004;114(5):1159‐1163. 10.1016/j.jaci.2004.08.007 15536425

[15] Flokstra‐de Blok BMJ , van der Meulen GN , DunnGalvin A , et al. Development and validation of the FAQL‐PB. Allergy. 2009;64(8):1209‐1217. 10.1111/j.1398-9995.2009.01968.x 19210345

[16] Moynihan D , Morrissey E , Soller L , et al. A short simple tool to measure the impact of food allergy on patients in routine clinical practice; the food allergy quality of life questionnaire, parent form 10 (FAQLQ‐PF10). Clin Transl Allergy. 2015;5(Suppl 3):P7.

[17] Indinnimeo L , Baldini L , De Vittori V , et al. Duration of a cow‐milk exclusion diet worsens parents' perception of quality of life in children with food allergies. BMC Pediatr. 2013;13:203. 10.1186/1471-2431-13-203 PMC4233620 24308381

[18] DunnGalvin A , Koman E , Raver E , et al. An examination of the food allergy quality of life questionnaire performance in a countrywide American sample of children: cross‐cultural differences in age and impact in the United States and Europe. J Allergy Clin Immunol Pract. 2017;5(2):363‐368. 10.1016/j.jaip.2016.09.049 28017626

[19] World Health Organization . Process of Translation and Adaptation of Instruments. World Health Organization; 2020 Accessed 15 Oct 2020. https://www.who.int/substance_abuse/research_tools/translation/en/.

[20] Varni JW , Seid M , Rode CA . The PedsQL: measurement model for pediatric quality of life. Med Care. 1999;37(2):126‐139. 10.1097/00005650-199902000-00003 10024117

[21] Bottesi G , Ghisi M , Altoè G , Conforti E , Melli G , Sica C . The Italian version of the depression anxiety stress Scales‐21: factor structure and psychometric properties on community and clinical samples. Compr Psychiatry. 2015;60:170‐181. 10.1016/j.comppsych.2015.04.005 25933937

[22] Ramos A , Warren C , Herbert L , et al. Validation of the food allergy quality of life questionnaire (FAQLQ‐PF10) short form in a diverse US sample. J Allergy Clin Immunol Pract. 2023;11(7):2239‐2241. 10.1016/j.jaip.2023.04.011 PMC10330312 37087096

[23] Kauppila TK , Kukkonen K , Pelkonen AS , Mäkelä MJ . The Finnish versions of food allergy quality of life questionnaires including parent form 10. Acta Paediatr. 2024;113(1):98‐104. 10.1111/apa.16995 37815009

[24] Ward C , Greenhawt M . Differences in caregiver food allergy quality of life between tertiary care, specialty clinic, and caregiver‐reported food allergic populations. J Allergy Clin Immunol Pract. 2016;4(2):257‐264.e3. 10.1016/j.jaip.2015.07.023 26433752

[25] Knibb RC , Stalker C . Validation of the food allergy quality of life‐parental burden questionnaire in the UK. Qual Life Res. 2013;22(7):1841‐1849. 10.1007/s11136-012-0295-3 23076799

[26] Norman G . Likert scales, levels of measurement and the ‘laws’ of statistics. Adv Health Sci Educ Theory Pract. 2010;15(5):625‐632. 10.1007/s10459-010-9222-y 20146096

[27] Polloni L , Ronconi L , Bonichini S , et al. Quality of life, mental health, and illness perception in pediatric food allergy. Children (Basel). 2025;12(12):1657. 10.3390/children12121657 PMC12731561 41462797

[28] Lavelle TA , Wittenberg E , Lamarand K , Prosser LA . Variation in the spillover effects of illness on parents, spouses, and children of the chronically ill. Appl Health Econ Health Policy. 2014;12(2):117‐124. 10.1007/s40258-014-0079-8 PMC4307398 24590611

[29] Howe L , Franxman T , Teich E , Greenhawt M . What affects quality of life among caregivers of food allergic children? Ann Allergy Asthma Immunol. 2014;113:69.e74. 10.1016/j.anai.2014.04.016 24950845

[30] Mizuno Y , Ohya Y , Nagao M , DunnGalvin A , Fujisawa T . Validation and reliability of the Japanese version of the food allergy quality of life questionnaire‐parent form. Allergol Int. 2017;66(2):290‐295. 10.1016/j.alit.2016.06.013 27599914

[31] Kubala SA , Young FD , Callier V , et al. Key factors that influence quality of life in patients with IgE‐mediated food allergy vary by age. Allergy. 2024;79(10):2812‐2825. 10.1111/all.16253 PMC11449640 39096008

[32] Canani RB , Nocerino R , Terrin G , Leone L , Troncone R . Hospital admissions for food‐induced anaphylaxis in Italian children. Clin Exp Allergy. 2012;42(12):1813‐1814. 10.1111/cea.12036 23181797

[33] Grabenhenrich LB , Dölle S , Moneret‐Vautrin A , et al. Anaphylaxis in children and adolescents: the European anaphylaxis registry. J Allergy Clin Immunol. 2016;137(4):1128‐1137.e1. 10.1016/j.jaci.2015.11.015 26806049

[34] Vetander M , Helander D , Flodström C , et al. Anaphylaxis and reactions to foods in children—A population‐based case study of emergency department visits. Clin Exp Allergy. 2012;42(4):568‐577. 10.1111/j.1365-2222.2011.03954.x 22417215

[35] Ward CE , Greenhawt MJ . Treatment of allergic reactions and quality of life among caregivers of food‐allergic children. Ann Allergy Asthma Immunol. 2015;114(4):312‐318.e2. 10.1016/j.anai.2014.12.022 25660416

