# Supplementary material for: Validation and reliability of the Italian versions of the Food Allergy Quality of Life ‐Parental Burden (FAQL‐PB) and ‐Parent Form 10 (FAQLQ‐PF10) Questionnaires
Source: Pediatr Allergy Immunol. 2026 Oct 2;37(10):e70510. doi: 10.1111/pai.70510 (PMC13633388; doi:10.1111/pai.70510)
Supplement: Supplementary file 1 [file PAI-37-e70510-s001.docx]

**Supplemental materials**

**Electronic Repository Files**

**Appendix S1. Cross-Cultural Adaptation from English to Italian of 2 food allergy-related questionnaires (FAQLPB and FAQLQ10-PF) according to WHO’s Guidelines [16]**

1. *Copyright and conditions of use*: The original developers of the questionnaire were contacted to notify the intention to undertake translation work and to agree to the translation methodology (i.e., approve the research protocol).
2. *Forward Translation - from English to Italian*: Two independent bilingual translators aimed at conceptual equivalence rather than a literal translation. One translator (JD) is a native English speaker with expertise in the translation of medical questionnaires working for the University of Pisa, and the other one (PC) is a medical doctor, native Italian speaker with expertise in food allergy and health-related quality of life terminology.
3. *Review of forward translation*: The two independent translators and a third member from the research team (i.e., a second healthcare professional (DP) native Italian speaker with expertise in food allergy and health-related quality of life terminology) reviewed and compared the two translations and agreed on a consensus version.
4. *Backward translation into English*: The agreed forward translation underwent backward translation into English by an independent native English translator blinded to the original questionnaires (Elsevier Translation Online Service). As in the initial translation, emphasis in the backtranslation was on conceptual and cultural equivalence rather than linguistic equivalence.
5. *Review of backward translation*: The original questionnaire and backward translations were reviewed for consistency and discrepancies by the main developers of the different questionnaires, who confirmed correct interpretation of conceptual content. Content validity was subsequently examined by three independent experts with no conflict of interest, who evaluated the relevance and representativeness of each item with respect to the constructs investigated.
6. *Pre-testing translated questionnaire*: Pre-testing of the preliminary Italian versions was performed in a sample of parents of children with food allergy. Cognitive interviews were conducted to evaluate comprehension, clarity, interpretation of individual items, and cultural relevance. Participants were asked to comment on the wording of each item, the response options, and whether any concepts were difficult to understand or not applicable to the Italian context. Feedback obtained during the interviews was reviewed by the research team, and minor linguistic modifications were introduced when required to improve conceptual equivalence while maintaining consistency with the original questionnaires.
7. *Face validity findings*. During pilot testing, parents generally reported that both questionnaires were easy to understand, relevant to their experience of managing childhood food allergy, and acceptable in terms of length and completion time. Minor comments mainly concerned wording preferences rather than conceptual issues. Based on participant feedback, small linguistic modifications were introduced to improve clarity and readability while preserving the conceptual equivalence of the original instruments. No items were considered inappropriate for the Italian cultural context, and no additional concepts were suggested by participants.
8. *Final questionnaires to be field-tested:* The final Italian versions of the FAQL-PB and FAQLQ-PF10 were administered to the target population for psychometric evaluation. A randomly selected subgroup of participants completed the questionnaires twice at a one-week interval, to minimize recall bias while assuming stability of the measured construct. The agreement between the two administrations was evaluated using intraclass correlation coefficients. Internal consistency was assessed using Cronbach’s alpha coefficients for total scores and subscales. Construct validity was examined by assessing convergent and discriminant validity. Convergent validity was evaluated by calculating Spearman correlation coefficients between the two translated questionnaires and a generic pediatric quality of life instrument (i.e. Pediatric Quality of Life Inventory, PedsQL™) a generic screening questionnaire assessing stress, anxiety, and depression in adults (i.e Depression Anxiety Stress Scales, DASS21) and a disease-specific instrument that assesses parental expectations regarding food allergy-related outcomes (i.e. Food Allergy Independent Measure, FAIM). Discriminant validity was assessed by comparing participants with and without food Allergy using an unpaired t-test.

**Appendix S2. Scoring of the FAQLQ-PF10 and FAQL-PB**

The total scores for the Food Allergy Quality of Life Questionnaire – Parent Form 10 (FAQLQ-PF10) and the Food Allergy Quality of Life – Parental Burden (FAQL-PB) were calculated as the mean of all item scores, according to the validated scoring procedures for the respective questionnaires. These total scores were used for all analyses presented in the main manuscript.

For exploratory purposes, factor-derived subdomain scores were also calculated as the mean of the items loading onto each factor identified by Kauppila et al (2024) for the FAQL-PF10^23^ and by Knibb et al (2013) for the FAQL-PB^25^. These exploratory subdomain scores were not used for the primary analyses reported in the manuscript.

The FAQLQ-PF10 comprises the following exploratory subdomains:

- Emotional Impact: Items 1, 8, and 9.
- Food Anxiety: Items 2, 3, 4, and 10.
- Social and Dietary Limitations: Items 5, 6, and 7.

The FAQL-PB comprises the following subdomains:

- Limitations in Life: Items 1, 2, 3, 4, 5, 15
- Emotional Distress: Items 6, 7, 8, 9, 10, 11, 12, 13, 14, 16, 17

Table S1. Summary of Cronbach’s α for the total scores and the exploratory factor-derived subdomains

| **FAQLQ-PF10** | Cronbach’s α |
| --- | --- |
| Total Score | 0.87 |
| Subdomain Emotional Impact | 0.74 |
| Subdomain Food Anxiety | 0.75 |
| Subscale Social Dietary Limitation | 0.75 |
| **FAQL-PB** |  |
| Total score | 0.95 |
| Subdomain Limitations on Life | 0.88 |
| Subdomain Emotional Distress | 0.94 |

Table S2. Summary of Convergent Validity (Spearman’s correlation coefficient) for the total scores and the exploratory factor-derived subdomains

| **FAQLQ-PF10** | FAQL-PB | FAIM  Total Score | PEDsQL Total Score | PEDsQL  Physical | PEDsQL  PsychoSocial |
| --- | --- | --- | --- | --- | --- |
| Total Score | **0.493*** | **0.550*** | -0.162 | 0.041 | **-0.232** |
| Subdomain Emotional Impact | **0.421*** | **0.543*** | -0.173 | -0.145 | **-0.202** |
| Subdomain Food Anxiety | **0.273*** | **0.320*** | -0.100 | 0.111 | -0.174 |
| Subdomain Social Dietary Limitation | **0.602*** | **0.639*** | -0.146 | -0.010 | **-0.182** |

| **FAQL-PB** | FAQLQ-PF10  Total Score | FAIM  Total Score | PEDsQL Total Score | PEDsQL  Physical | PEDsQL  PsychoSocial |
| --- | --- | --- | --- | --- | --- |
| Total score | **0.493*** | **0.657*** | **-0.264*** | **-0.207** | **-0.228** |
| Subdomain Limitations on Life | **0.521*** | **0.637*** | **-0.220** | **-0.247** | -0.159 |
| Subdomain Emotional Distress | **0.493*** | **0.607*** | **-0.232*** | -0.137 | **-0.211*** |

*Significant p-values (p < 0.05) are shown in bold. * p < 0.01*

Table S3. Convergent Validity (Spearman’s correlation coefficient) between FAQLQ-PF10 and FAIM (children 0-12 y)

|  | **FAIM** | | | | | | | | **FAIM Subscales** | |
| --- | --- | --- | --- | --- | --- | --- | --- | --- | --- | --- |
| **FAQLQ-PF10** | Item 1 | Item 2 | Item 3 | Item 4 | Item 5 | Item 6 | Item 7 | Total Score | Expectation | Independent Measure |
| Item 1 | 0,020 | **0,387*** | **0,455*** | **0,423*** | **0,324*** | **0,572*** | **0,468*** | **0,575*** | **0,470*** | **0,543*** |
| Item 2 | -0,185 | 0,121 | **0,255*** | **0,250** | **0,307*** | **0,301*** | 0,181 | **0,276*** | 0,188 | **0,312*** |
| Item 3 | -0,189 | 0,159 | **0,200** | **0,194** | 0,179 | **0,344*** | **0,228** | **0,262*** | 0,148 | **0,305*** |
| Item 4 | 0,016 | **0,191** | **0,238** | 0,189 | **0,504*** | **0,444*** | **0,389*** | **0,441*** | **0,261*** | **0,516*** |
| Item 5 | 0,117 | **0,478*** | **0,494*** | **0,306*** | **0,534*** | **0,641*** | **0,594*** | **0,679*** | **0,504*** | **0,690*** |
| Item 6 | 0,099 | **0,235** | **0,306*** | **0,392*** | **0,276*** | **0,412*** | **0,336*** | **0,447*** | **0,378*** | **0,412*** |
| Item 7 | 0,038 | **0,282*** | **0,348*** | **0,444*** | **0,305*** | **0,502*** | **0,363*** | **0,479*** | **0,393*** | **0,458*** |
| Item 8 | **-0,319*** | 0,125 | **0,254*** | 0,136 | 0,123 | **0,233** | 0,154 | 0,163 | 0,109 | **0,199** |
| Item 9 | **-0,210** | 0,064 | 0,154 | 0,192 | 0,112 | 0,191 | 0,055 | 0,128 | 0,091 | 0,144 |
| Item 10 | -0,031 | 0,149 | **0,291*** | **0,338*** | 0,150 | **0,243** | 0,152 | **0,278*** | **0,276*** | **0,207** |
| Total Score | -0,080 | **0,331*** | **0,452*** | **0,423*** | **0,388*** | **0,568*** | **0,421*** | **0,550*** | **0,425*** | **0,548*** |
| Subdomain Emotional Impact | 0,044 | **0,314*** | **0,375*** | **0,471*** | **0,329*** | **0,543*** | **0,413*** | **0,543*** | **0,454*** | **0,507*** |
| Subdomain Food Anxiety | **-0,184** | 0,160 | **0,337*** | **0,299*** | **0,238*** | **0,326*** | **0,238*** | **0,320*** | **0,259*** | **0,311*** |
| Subdomain Social Dietary Limitation | -0,045 | **0,395*** | **0,481*** | **0,395*** | **0,502*** | **0,612*** | **0,516*** | **0,639*** | **0,473*** | **0,645*** |

*Significant p-values (p < 0.05) are shown in bold. * p < 0.01*

Table S4. Convergent Validity (Spearman’s correlation coefficient) between FAQL-PB (17-items) and FAIM

|  | **FAIM** | | | | | | | | **FAIM Subscales** | |
| --- | --- | --- | --- | --- | --- | --- | --- | --- | --- | --- |
| **FAQL-PB** | Item 1 | Item 2 | Item 3 | Item 4 | Item 5 | Item 6 | Item 7 | Total Score | Expectation | Independent Measure |
| Item 1 | 0,040 | **0,474*** | **0,477*** | **0,271*** | **0,492*** | **0,519*** | **0,536*** | **0,591*** | **0,455*** | **0,588*** |
| Item 2 | 0,140 | **0,364*** | **0,328*** | **0,220** | **0,480*** | **0,577*** | **0,561*** | **0,560*** | **0,371*** | **0,625*** |
| Item 3 | **0,203** | **0,442*** | **0,488*** | **0,365*** | **0,452*** | **0,550*** | **0,529*** | **0,632*** | **0,524*** | **0,586*** |
| Item 4 | **0,234** | **0,314*** | **0,274*** | 0,164 | **0,329*** | **0,412*** | **0,493*** | **0,449*** | **0,341*** | **0,478*** |
| Item 5 | **0,213** | **0,252*** | **0,303*** | **0,230** | **0,282*** | **0,417*** | **0,477*** | **0,446*** | **0,345*** | **0,452*** |
| Item 6 | 0,020 | **0,369*** | **0,399*** | 0,164 | **0,375*** | **0,392*** | **0,399*** | **0,433*** | **0,342*** | **0,442*** |
| Item 7 | 0,034 | **0,431*** | **0,358*** | **0,291*** | **0,473*** | **0,438*** | **0,432*** | **0,517*** | **0,409*** | **0,509*** |
| Item 8 | 0,107 | **0,364*** | **0,376*** | **0,297*** | **0,426*** | **0,503*** | **0,433*** | **0,522*** | **0,404*** | **0,518*** |
| Item 9 | 0,083 | **0,442*** | **0,508*** | **0,314*** | **0,384*** | **0,518*** | **0,420*** | **0,569*** | **0,502*** | **0,500*** |
| Item 10 | 0,048 | **0,374*** | **0,408*** | **0,345*** | **0,447*** | **0,471*** | **0,446*** | **0,527*** | **0,426*** | **0,527*** |
| Item 11 | 0,088 | **0,446*** | **0,450*** | **0,368*** | **0,349*** | **0,535*** | **0,446*** | **0,579*** | **0,485*** | **0,507*** |
| Item 12 | -0,010 | **0,423*** | **0,450*** | **0,257*** | **0,369*** | **0,471*** | **0,492*** | **0,514*** | **0,402*** | **0,508*** |
| Item 13 | 0,023 | **0,290*** | **0,264*** | **0,274*** | **0,312*** | **0,317*** | **0,375*** | **0,381*** | **0,293*** | **0,368*** |
| Item 14 | 0,096 | **0,390*** | **0,391*** | **0,307*** | **0,476*** | **0,491*** | **0,503*** | **0,554*** | **0,421*** | **0,564*** |
| Item 15 | -0,079 | **0,197** | **0,180*** | 0,136 | **0,412*** | **0,400*** | **0,412*** | **0,336*** | 0,160 | **0,463*** |
| Item 16 | 0,046 | **0,357*** | **0,271*** | 0,178 | **0,378*** | **0,454*** | **0,429*** | **0,448*** | **0,297*** | **0,489*** |
| Item 17 | -0,032 | **0,324*** | **0,217** | **0,217** | **0,270*** | **0,330*** | **0,298*** | **0,321*** | **0,250*** | **0,339*** |
| Total Score | 0,081 | **0,504*** | **0,484*** | **0,354*** | **0,527*** | **0,598*** | **0,589*** | **0,657*** | **0,510*** | **0,652*** |
| Subdomain Limitations on Life | 0.098 | **0.448*** | **0.438*** | **0.293*** | **0.536*** | **0.612*** | **0.640*** | **0.637*** | **0.454*** | **0.685*** |
| Subdomain Emotional Distress | 0.027 | **0.484*** | **0.473*** | **0.343*** | **0.481*** | **0.553*** | **0.527*** | **0.607*** | **0.477*** | **0.593*** |

*Significant p-values (p < 0.05) are shown in bold. * p < 0.01*

Table S5. Convergent Validity (Spearman’s coefficient) between FAQL-PB (17-items) and FAQLQ-PF10 (10 items and 3 subscales) (children 0-12 y)

|  | **FAQLQ-PF10** | | | | | | | | | | | **FAQLQ-PF10 Subscales** | | |
| --- | --- | --- | --- | --- | --- | --- | --- | --- | --- | --- | --- | --- | --- | --- |
| **FAQL-PB** | Item 1 | Item 2 | Item 3 | Item 4 | Item 5 | Item 6 | Item 7 | Item 8 | Item 9 | Item 10 | Total Score | Emotional Impact | Food  Anxiety | Social Dietary Limitation |
| Item 1 | **0,372*** | **0,284*** | **0,354*** | **0,386*** | **0,776*** | **0,404*** | **0,424*** | **0,208** | **0,249** | **0,220** | **0,549*** | **0,431*** | **0,309*** | **0,608*** |
| Item 2 | **0,377*** | **0,247** | **0,350*** | **0,387*** | **0,800*** | **0,415*** | **0,443*** | 0,156 | 0,143 | 0,160 | **0,505*** | **0,461*** | **0,219** | **0,577*** |
| Item 3 | **0,427*** | **0,283*** | **0,266*** | **0,408*** | **0,746*** | **0,442*** | **0,447*** | 0,155 | 0,156 | **0,234** | **0,526*** | **0,479*** | **0,274*** | **0,580*** |
| Item 4 | **0,276*** | 0,013 | 0,037 | **0,240** | **0,432*** | 0,163 | **0,191** | -0,033 | -0,001 | -0,061 | **0,212** | **0,217** | 0,006 | **0,300*** |
| Item 5 | **0,337*** | **0,193** | 0,118 | **0,289*** | **0,490*** | **0,238** | **0,375*** | -0,045 | -0,007 | 0,107 | **0,313*** | **0,321*** | 0,044 | **0,360*** |
| Item 6 | **0,239** | **0,251*** | 0,164 | **0,296*** | **0,437*** | 0,081 | **0,263*** | 0,156 | 0,083 | 0,003 | **0,292*** | **0,199** | 0,173 | **0,386*** |
| Item 7 | **0,288*** | **0,217** | 0,182 | **0,415*** | **0,470*** | **0,203** | **0,373*** | 0,074 | 0,134 | 0,102 | **0,353*** | **0,315*** | **0,192** | **0,442*** |
| Item 8 | **0,339*** | 0,163 | 0,142 | **0,387*** | **0,422*** | **0,213** | **0,367*** | **0,210** | 0,131 | 0,134 | **0,373*** | **0,373*** | **0,256*** | **0,429*** |
| Item 9 | **0,410*** | **0,217** | **0,221** | **0,342*** | **0,518*** | **0,356*** | **0,413*** | **0,336*** | **0,196** | 0,137 | **0,470*** | **0,447*** | **0,304*** | **0,488*** |
| Item 10 | **0,421*** | **0,249*** | **0,303*** | **0,347*** | **0,561*** | **0,290*** | **0,366*** | **0,221** | **0,267*** | 0,044 | **0,455*** | **0,385*** | **0,247*** | **0,557*** |
| Item 11 | **0,367*** | **0,245** | **0,306*** | **0,416*** | **0,570*** | **0,302*** | **0,446*** | **0,217** | **0,199** | 0,165 | **0,496*** | **0,431*** | **0,262*** | **0,528*** |
| Item 12 | **0,323*** | **0,232** | **0,270*** | **0,306*** | **0,498*** | 0,180 | **0,310*** | 0,157 | **0,224** | 0,039 | **0,370*** | **0,313*** | **0,242*** | **0,477*** |
| Item 13 | **0,193** | 0,129 | 0,127 | **0,224** | **0,308*** | 0,126 | **0,197** | 0,142 | 0,191 | 0,050 | **0,239** | **0,213** | **0,212** | **0,294*** |
| Item 14 | **0,339*** | **0,211** | 0,184 | **0,419*** | **0,560*** | **0,243** | **0,352*** | 0,039 | 0,183 | 0,031 | **0,372*** | **0,355*** | 0,174 | **0,511*** |
| Item 15 | 0,187 | 0,116 | 0,091 | **0,365*** | **0,408*** | 0,124 | 0,163 | -0,045 | 0,017 | -0,017 | **0,190** | **0,215** | 0,061 | **0,360*** |
| Item 16 | **0,247** | 0,158 | **0,221** | **0,407*** | **0,560*** | 0,177 | **0,239** | 0,075 | 0,101 | 0,106 | **0,344*** | **0,266*** | **0,194** | **0,466*** |
| Item 17 | 0,158 | 0,083 | 0,164 | **0,197** | **0,326*** | 0,003 | 0,092 | 0,124 | **0,200** | 0,000 | **0,208** | 0,100 | 0,163 | **0,287*** |
| Total Score | **0,405*** | **0,262*** | **0,283*** | **0,458*** | **0,688*** | **0,303*** | **0,413*** | 0,179 | **0,216** | 0,121 | **0,493*** | **0,421*** | **0,273*** | **0,602*** |
| Subdomain Limitations on Life | **0.437*** | **0.245*** | **0.296*** | **0.433*** | **0.780*** | **0.421*** | **0.422*** | **0.130*** | **0.155*** | **0.228*** | **0.521*** | **0.459*** | **0.235*** | **0.609*** |
| Subdomain Emotional Distress | **0.426*** | **0.254*** | **0.293*** | **0.439*** | **0.598*** | **0.310*** | **0.409*** | **0.233*** | **0.213** | **0.192** | **0.493*** | **0.389*** | **0.287*** | **0.557*** |

*Significant p-values (p < 0.05) are shown in bold. * p < 0.01*

Table S6. Convergent Validity (Spearman’s correlation coefficient) between FAQL-PB, PedsQL, and DASS21 scores

|  | **PedsQL** | **PedsQL Subscales** | | **DASS21 Subscales** | | |
| --- | --- | --- | --- | --- | --- | --- |
| **FAQL-PB** | Total Score | Physical | PsychoSocial | Stress | Anxiety | Depression |
| Item 1 | -0,094 | -0,127 | -0,074 | **0,264*** | 0,174 | **0,268*** |
| Item 2 | -0,126 | **-0,182** | -0,086 | **0,254*** | **0,183** | **0,280*** |
| Item 3 | -0,066 | -0,119 | -0,033 | **0,184*** | 0,080 | **0,203** |
| Item 4 | **-0,280*** | **-0,269*** | **-0,224** | **0,252*** | 0,138 | **0,280*** |
| Item 5 | **-0,245*** | **-0,303*** | -0,165 | **0,258*** | 0,156 | **0,180** |
| Item 6 | -0,116 | -0,077 | -0,068 | **0,321*** | 0,174 | **0,257*** |
| Item 7 | **-0,249*** | **-0,179** | **-0,222** | **0,341*** | **0,231** | **0,330*** |
| Item 8 | **-0,189** | -0,167 | -0,147 | **0,233** | 0,174 | **0,240*** |
| Item 9 | -0,176 | -0,073 | **-0,185** | **0,231** | **0,183** | **0,243*** |
| Item 10 | **-0,216** | -0,128 | **-0,206** | **0,369*** | **0,217** | **0,319*** |
| Item 11 | -0,156 | -0,093 | -0,153 | **0,270*** | 0,161 | **0,268*** |
| Item 12 | **-0,212** | -0,109 | **-0,212** | **0,320*** | **0,253*** | **0,265*** |
| Item 13 | **-0,227** | -0,159 | **-0,181** | **0,287*** | 0,178 | **0,198** |
| Item 14 | **-0,227** | **-0,189** | **-0,198** | **0,357*** | **0,188** | **0,368*** |
| Item 15 | **-0,278*** | **-0,289*** | **-0,210** | 0,125 | 0,176 | **0,228** |
| Item 16 | **-0,258*** | **-0,180** | **-0,242*** | **0,229** | 0,160 | **0,225** |
| Item 17 | -0,175 | -0,039 | -0,175 | **0,214** | 0,097 | 0,116 |
| Total Score | **-0,264*** | **-0,207** | **-0,228** | **0,342*** | **0,222** | **0,324*** |
| Subdomain Limitations on Life | **-0.220** | **-0.247*** | -0.159 | **0.270*** | **0.197** | **0.328*** |
| Subdomain Emotional Distress | **-0.232** | -0.137 | **-0.211** | **0.354*** | **0.238*** | **0.318*** |

Significant p-values (p < 0.05) are shown in bold. * p < 0.01

Table S7. Convergent Validity (Spearman’s correlation coefficient) between FAQLQ-PF10, PedsQL, and DASS21 scores (children 0-12 y)

|  | **PedsQL** | **PedsQL Subscales** | | **DASS21 Subscales** | | |
| --- | --- | --- | --- | --- | --- | --- |
| **FAQLQ-PF10** | Total Score | Physical | PsychoSocial | Stress | Anxiety | Depression |
| Item 1 | -0,159 | 0,034 | **-0,235** | **0,356*** | **0,241** | **0,255*** |
| Item 2 | -0,172 | 0,044 | **-0,228** | **0,330*** | **0,211** | **0,218** |
| Item 3 | -0,171 | 0,056 | **-0,260*** | **0,267*** | **0,250** | **0,301*** |
| Item 4 | -0,146 | -0,127 | -0,117 | **0,321*** | **0,242** | **0,334*** |
| Item 5 | 0,015 | -0,054 | 0,065 | **0,220** | 0,190 | **0,241** |
| Item 6 | -0,164 | -0,106 | **-0,196** | 0,170 | 0,118 | **0,211** |
| Item 7 | -0,175 | -0,138 | **-0,203** | **0,272*** | **0,250** | **0,247** |
| Item 8 | -0,008 | **0,229** | -0,126 | 0,122 | 0,039 | 0,063 |
| Item 9 | -0,105 | 0,079 | -0,161 | 0,033 | 0,036 | -0,013 |
| Item 10 | -0,073 | 0,103 | -0,134 | 0,086 | 0,085 | 0,040 |
| Total Score | -0,162 | 0,041 | **-0,232** | **0,320*** | **0,236** | **0,268*** |
| Subdomain Emotional Impact | -0,173 | -0,145 | **-0,202** | **0,201** | **0,204** | **0,234** |
| Subdomain Food Anxiety | -0,100 | 0,111 | -0,174 | 0,102 | 0,103 | 0,078 |
| Subdomain Social Dietary Limitation | -0,146 | -0,010 | **-0,182** | **0,354*** | **0,280*** | **0,355*** |

*Significant p-values (p < 0.05) are shown in bold. * p < 0.01*

Table S8. Discriminant Validity for the FAQL-PB and FAQLQ-PF10 questionnaires between children with food allergy (n=128) and healthy controls (n=52).

| **FAQL-PB** | **Food Allergy** | **Controls** | ***p-value*** |
| --- | --- | --- | --- |
| Item 1 | 2.56 ± 1.98 | 0.08 ± 0.33 | **< 0.0001** |
| Item 2 | 2.46 ± 2.06 | 0.04 ± 0.28 | **< 0.0001** |
| Item 3 | 2.50 ± 1.87 | 0.02 ± 0.14 | **< 0.0001** |
| Item 4 | 1.43 ± 1.58 | 0 ± 0 | **< 0.0001** |
| Item 5 | 1.52 ± 1.64 | 0 ± 0 | **< 0.0001** |
| Item 6 | 2.60 ± 1.96 | 0 ± 0 | **< 0.0001** |
| Item 7 | 3.4 ± 1.78 | 0 ± 0 | **< 0.0001** |
| Item 8 | 2.98 ± 1.82 | 0 ± 0 | **< 0.0001** |
| Item 9 | 3.02 ± 2.03 | 0 ± 0 | **< 0.0001** |
| Item 10 | 2.98 ± 2.06 | 0 ± 0 | **< 0.0001** |
| Item 11 | 2.98 ± 1.89 | 0.02 ± 0.14 | **< 0.0001** |
| Item 12 | 3.36 ± 1.87 | 0 ± 0 | **< 0.0001** |
| Item 13 | 3.66 ± 1.98 | 0 ± 0 | **< 0.0001** |
| Item 14 | 2.59 ± 2.03 | 0 ± 0 | **< 0.0001** |
| Item 15 | 2.17 ± 1.93 | 0 ± 0 | **< 0.0001** |
| Item 16 | 3.06 ± 1.95 | 0 ± 0 | **< 0.0001** |
| Item 17 | 3.84 ± 1.82 | 0 ± 0 | **< 0.0001** |
| Total Score | 2.77 ± 1.43 | 0.01 ± 0.05 | **< 0.0001** |
| Subdomain Limitations on Life | 2.08 ± 1.48 | 0.02 ± 0.12 | **< 0.0001** |
| Subdomain Emotional Distress | 3.10 ± 1.57 | 0.00 ± 0.01 | **< 0.0001** |
| **FAQLQ-PF10** | **Food Allergy** | **Controls** | ***p-value*** |
| Item 1 | 2.35 ± 1.91 | 0 ± 0 | **< 0.0001** |
| Item 2 | 2.56 ± 2.08 | 0.10 ± 0.3 | **< 0.0001** |
| Item 3 | 2.01 ± 1.87 | 0.02 ± 0.14 | **< 0.0001** |
| Item 4 | 3.33 ± 1.85 | 0.1 ± 0.57 | **< 0.0001** |
| Item 5 | 2.75 ± 2.22 | 0 ± 0 | **< 0.0001** |
| Item 6 | 2.04 ± 2.03 | 0 ± 0 | **< 0.0001** |
| Item 7 | 1.70 ± 1.82 | 0 ± 0 | **< 0.0001** |
| Item 8 | 4.03 ± 2.14 | 0 ± 0 | **< 0.0001** |
| Item 9 | 4.57 ± 2.01 | 0 ± 0 | **< 0.0001** |
| Item 10 | 2.71 ± 1.98 | 0.06 ± 0.42 | **< 0.0001** |
| Total Score | 2.79 ± 1.36 | 0.03 ± 0.08 | **< 0.0001** |
| Subdomain Emotional Impact | 1.74 ± 1.69 | 0 ± 0 | **< 0.0001** |
| Subdomain Food Anxiety | 3.67 ± 1.77 | 0.02 ± 0.15 | **< 0.0001** |
| Subdomain Social Dietary Limitation | 2.55 ± 1.46 | 0.03 ± 0.13 | **< 0.0001** |

Table S9. Univariate Regression Analysis for FAQL-PB Mean Total Score and FAQLQ-10 Mean Total Score

|  | **FAQL-PB Total Score** | | | | **FAQLQ-PF10 Total Score** | | | |
| --- | --- | --- | --- | --- | --- | --- | --- | --- |
| **Characteristics** | **Coefficient** | **p-value** | **95%CI** | | **Coefficient** | **p-value** | **95%CI** | |
| **Children Age** | -0.065 | 0.388 | -0.213 | 0.083 | 0.065 | **0.025** | 0.008 | 0.122 |
| Children Age Groups   - 0-3 years | 0.090 | 0.420 | -0.129 | 0.308 | -0.623 | 0.141 | -1.456 | 0.209 |
| - 4-6 years | 0.170 | 0.109 | -0.038 | 0.379 | 0.463 | 0.299 | -0.415 | 1.340 |
| - 7-12 years | 0.021 | 0.850 | -0.201 | 0.244 | 0.143 | 0.732 | -0.678 | 0.963 |
| - 13-17 years | 0.000 | 0.000 |  |  | 0.000 | 0.141 | -1.456 | 0.209 |
| Children Gender (Male) | 0.041 | 0.879 | -0.486 | 0.568 | -0.032 | 0.906 | -0.566 | 0.502 |
| **Allergic comorbidities** | 1.569 | **<0.0001** | 1.109 | 2.029 | 1.791 | **<0.0001** | 1.349 | 2.233 |
| ≥1 Siblings | -0.044 | 0.562 | -0.193 | 0.105 | 0.291 | 0.294 | -0.255 | 0.836 |
| **Other siblings with Food Allergy** | 0.092 | 0.229 | -0.058 | 0.241 | 1.542 | **0.009** | 0.398 | 2.686 |
| Caregiver age | -0.009 | 0.906 | -0.160 | 0.141 | 0.012 | 0.428 | -0.018 | 0.043 |
| Caregiver Education level   - Middle School | 0.000 | 0.000 |  |  | 0.000 | 0.000 |  |  |
| - High School | -0.028 | 0.830 | -0.284 | 0.229 | -0.015 | 0.973 | -0.895 | 0.864 |
| - Bachelor | -0.113 | 0.361 | -0.356 | 0.130 | -0.133 | 0.781 | -1.077 | 0.811 |
| - Master | -0.067 | 0.526 | -0.274 | 0.141 | -0.158 | 0.778 | -1.261 | 0.945 |
| **Caregiver Marital Status**   - **Unmarried** | -0.609 | **0.035** | -1.175 | -0.043 | -2.079 | 0.227 | -5.463 | 1.304 |
| - Married | -0.573 | 0.072 | -1.198 | 0.052 | -1.555 | 0.361 | -4.902 | 1.793 |
| - Divorced | -0.367 | 0.058 | -0.747 | 0.012 | -1.483 | 0.400 | -4.954 | 1.987 |
| - Widowed | 0.000 | 0.000 |  |  | 0.000 | 0.000 |  |  |
| Household Income (Euros/y)   - <25.000 | -0.171 | 0.422 | -0.591 | 0.249 | -0.493 | 0.544 | -2.098 | 1.111 |
| - 25.000-50.000 | -0.077 | 0.741 | -0.535 | 0.382 | -0.090 | 0.910 | -1.655 | 1.476 |
| - 50.000-100.000 | 0.185 | 0.773 | -0.418 | 0.312 | 0.250 | 0.767 | -1.412 | 1.912 |
| - >100.000 | 0.000 | 0.000 |  |  | 0.000 | 0.000 |  |  |
| Caregiver Chronic Diseases | 0.134 | 0.679 | -0.499 | 0.766 | 0.111 | 0.731 | -0.522 | 0.744 |
| **Main Food Allergy (Milk)** | 30.429 | **0.014** | 0.087 | 0.772 | 0.535 | 0.273 | -0.427 | 1.497 |
| **Milk or Egg vs Peanut or Tree nut** | -0.277 | **0.002** | -0.447 | -0.107 | -0.235 | 0.347 | -0.729 | 0.258 |
| Multiple Food Allergy | 0.111 | 0.213 | -0.065 | 0.287 | 0.382 | 0.126 | -0.109 | 0.872 |
| **Years of Elimination Diet** | -0.024 | 0.791 | -0.201 | 0.153 | 0.105 | **0.0004** | 0.048 | 0.161 |
| **Anaphylaxis** | 0.179 | **0.049** | 0.000 | 0.357 | 0.820 | **0.001** | 0.343 | 1.297 |
| History of Epinephrine use | 0.041 | 0.651 | -0.139 | 0.221 | 0.357 | 0.453 | -0.582 | 1.296 |
| **Emergency Department Visit for food allergy** | 0.185 | **0.047** | 0.003 | 0.367 | 0.686 | **0.012** | 0.151 | 1.221 |
| Oral Food Challenge in the past | 0.027 | 0.761 | -0.150 | 0.204 | 0.346 | 0.161 | -0.139 | 0.832 |

Table S10. Multivariable ordinal logistic regression analysis for variables predictive of FAQL-PB Total Score and FAQLQ-PF10 Total Score

| **FAQL-PB Total Score** | | | | | |
| --- | --- | --- | --- | --- | --- |
|  | Coefficient | *p*-value | | 95%-CI | |
| Anaphylaxis | 0.89 | **0.007** | | 0.246 — 1.533 | |
| Allergic comorbidities | / | / | | / | |
| Emergency Department Visit for Food Allergy | / | / | | / | |
| Main food allergy | / | / | | / | |
| Milk or Egg vs Peanut or Tree nuts | 1.08 | **0.001** | | 0.429 — 1.733 | |
| **FAQLQ-PF10 Total Score** | | | | | |
|  | Coefficient | | *p*-value | | 95%-CI |
| Anaphylaxis | 0.91 | | **0.023** | | 0.125 — 1.692 |
| Allergic comorbidities | 0.30 | | 0.421 | | 0.432 — 1.033 |
| Emergency Department Visit for food allergy | 0.45 | | 0.275 | | 0.357 — 1.254 |
| Children age | 0.12 | | **0.006** | | 0.033 — 0.205 |
| Years of elimination diet | / | | / | | / |
| Other siblings with food allergy | 0.460 | | 0.536 | | 1.005 — 1.932 |
